# Comprehensive genome analysis of a pangolin-associated *Paraburkholderia fungorum* provides new insights into its secretion systems and virulence

**DOI:** 10.7717/peerj.9733

**Published:** 2020-09-03

**Authors:** Ka Yun Tan, Avirup Dutta, Tze King Tan, Ranjeev Hari, Rofina Y. Othman, Siew Woh Choo

**Affiliations:** 1 Institute of Biological Sciences, Faculty of Science, Universiti Malaya, Kuala Lumpur, Malaysia; 2 Genome Informatics Research Laboratory, Centre for Research in Biotechnology for Agriculture (CEBAR), High Impact Research Building, Universiti Malaya, Kuala Lumpur, Malaysia; 3 Centre for Research in Biotechnology for Agriculture (CEBAR), Level 3, Research Management & Innovation Complex, Universiti Malaya, Copenhagen, Kuala Lumpur, Malaysia; 4 College of Science and Technology, Wenzhou-Kean University, Wenzhou, Zhejiang Province, China; 5 Current affiliation: The Novo Nordisk Foundation Center for Basic Metabolic Research, Human Genomics and Metagenomics in Metabolism, Faculty of Health and Medical Sciences, University of Copenhagen, Copenhagen, Denmark; 6 Current affiliation: Cancer Science Institute of Singapore, National University of Singapore, Singapore

**Keywords:** Secretion system, Taxonomy, Burkholderia, RAST

## Abstract

**Background:**

*Paraburkholderia fungorum (P. fungorum)* is a Gram-negative environmental species that has been commonly used as a beneficial microorganism in agriculture as an agent for biocontrol and bioremediation. Its use in agriculture is controversial as many people believe that it could harm human health; however, there is no clear evidence to support.

**Methodology:**

The pangolin *P. fungorum* (pangolin Pf) genome has a genomic size of approximately 7.7 Mbps with N50 of 69,666 bps. Our study showed that pangolin Pf is a *Paraburkholderia fungorum* supported by evidence from the core genome SNP-based phylogenetic analysis and the ANI analysis. Functional analysis has shown that the presence of a considerably large number of genes related to stress response, virulence, disease, and defence. Interestingly, we identified different types of secretion systems in the genome of pangolin Pf, which are highly specialized and responsible for a bacterium’s response to its environment and in physiological processes such as survival, adhesion, and adaptation. The pangolin Pf also shared some common virulence genes with the known pathogenic member of the Burkholderiales. These genes play important roles in adhesion, motility, and invasion.

**Conclusion:**

This study may provide better insights into the functions, secretion systems and virulence of this pangolin-associated bacterial strain. The addition of this genome sequence is also important for future comparative analysis and functional work of *P. fungorum.*

## Introduction

The genus *Burkholderia* is a group of Gram-negative, motile, obligate aerobic rod-shaped bacteria. Some members of the Burkholderiales species are environmentally important and some are pathogenic (Landers et al., 2000). The genus consists of more than 120 species spreading across soil and water environments that can be utilized in bioremediation and bioconversion application (Morya, Salvachúa & Thakur, 2020), while some being opportunistic pathogens of plants or humans (Estrada-de los Santos et al., 2013). The most famous among the pathogenic members of *Burkholderia* are *B . mallei* and *B . pseudomallei*. The *B . mallei* can cause glanders in horses, donkeys, and humans (Whitlock, Estes & Torres, 2007), whereas the *B . pseudomallei* can cause melioidosis in humans and animals (Limmathurotsakul et al., 2016). Another group of Burkholderia, *Burkholderia cepacia complex* (Bcc) is a group comprising at least 20 species which are known to be opportunistic pathogens in immunocompromised patients, especially those suffering cystic fibrosis (Rojas-Rojas et al., 2019; Leitão et al., 2017).

*Paraburkholderia* has been split from the *Burkholderia* genera (Sawana, Adeolu & Gupta, 2014; Beukes et al., 2017; Estrada-de Los Santos et al., 2018). It gained considerable importance for their abilities to fix nitrogen, promote plant growth, and degrade recalcitrant chemical compounds. It has been sustainable as alternatives to chemical fertilizers in agriculture, and also bioremediation of the impacted environment (Andreolli et al., 2011; Kaur, Selvakumar & Ganeshamurthy, 2017). *P. fungorum* can be isolated from diverse ecological niches including samples from human, animals, and plants (Coenye et al., 2001). Although previous studies reported the isolation of *P. fungorum* in synovial tissue of an overweight but healthy patient (Loong et al., 2019), and has also been shown to cause septicemia (Gerrits et al., 2005), and infectious granuloma (Zhang et al., 2014), no clear evidence for its pathogenicity has been reported. However, there are debates on its suitability as an agent of biodegradation and bioremediation because many people believe that it could affect human health (Jones, Dodd & Webb, 2001; Nally et al., 2018).

In a previous genome sequencing project, we sequenced the genomes of the infected cerebellum and cerebrum of a female pregnant Malayan pangolin (*Manis javanica)* and its infected fetus (Tan et al., 2016b; Choo et al., 2016b). A bacterium genome was identified in the pangolin genome data and identified as a *Paraburkholderia* species (hereto referred to as pangolin Pf). In this study, the pangolin Pf genome was further analysed to advance our understanding of its functions and virulence.

## Materials & Methods

### Genome datasets

The genome sequence of pangolin Pf (accession numbers: CP028829–CP028832) can be downloaded from the GenBank database at the National Center for Biotechnology Information (NCBI) website. It was assembled from the previous pangolin genome data (accession numbers: SRR3949798, SRR3949787, SRR3949728, SRR3949765, and SRR11935558) generated from the genome sequencing of an infected female Malayan pangolin (Tan et al., 2016b; Choo et al., 2020). Briefly, the raw reads generated from three *P. fungorum*-infected tissues (cerebrum, cerebellum and fetus) of the Malayan pangolin (manuscript in preparation) were pooled and assembled by mapping the reads to the reference genome, *Paraburkholderia fungorum* ATCC BAA-463 (Accession numbers: CP010024–CP010027) downloaded from NCBI using CLC Assembly Cell software with default parameters (D’Cruze et al., 2011). The mapped reads were assembled into a draft genome sequence for downstream analyses (Table 1).

### Genome annotation

The pangolin Pf genome sequence was submitted to the Rapid Annotation using Subsystem Technology (RAST) server for gene prediction and functional annotation (Aziz et al., 2008). To ensure the uniformity in the annotations for comparative analysis, the genome sequences of 17 closely related species (Table 1), were retrieved from NCBI (http://www.ncbi.nlm.nih.gov) (Pruitt, Tatusova & Maglott, 2007) and annotated by the RAST server.

### The core-genome SNP-based phylogenetic inference

The single nucleotide polymorphism (SNP) refers to a single-base variation in a DNA sequence (Vignal et al., 2002). The SNPs are the most abundant genetic variation, in a region that can be used to assess bacterial diversity and for taxonomic classification. The core-genome SNP-based phylogenetic analyses have successfully been used to infer the taxonomic relationships of many bacterial species (Ang et al., 2016; Choo et al., 2016a; Choo et al., 2014; Zheng et al., 2017). PanSeq was used to align all genome sequences and SNPs were identified in the conserved genomic regions among all species (Laing et al., 2010). All SNPs were extracted and concatenated into a long sequence for each genome. Phylogenetic tree based on the core-genome SNP was constructed using the genome sequences of the pangolin Pf and 17 selected closely related species (Table 1). The tree was constructed using MEGA-X (Kumar et al., 2018). Neighbour-joining tree was inferred using the Kimura’s two parameter model and nodal support was estimated using 1,000 replicates.

### Whole-genome Average Nucleotide Identity (ANI) analysis

To evaluate the genetic relatedness between the genome of pangolin Pf and its closely related species, the ANI values were calculated based on the method as previously described (Ang et al., 2016; Rodriguez & Konstantinidis, 2014; Goris et al., 2007; Tan et al., 2016a). Two-way BLAST was chosen and only the forward and reversed-matched orthologs were used in the calculations. For robustness, the BLAST match was set for at least 50% identity at the nucleotide and amino acid level and a sequence coverage of at least 70%.

### Genome Island and prophage prediction

All putative genomic islands (GIs) in the pangolin Pf genome were predicted using IslandViewer4, which is based on unique features in codon usage, dinucleotide sequence composition and the presence of mobile element genes (Bertelli et al., 2017; Langille & Brinkman, 2009). The RAST provided GenBank file was submitted into IslandViewer4 web server for GI prediction.

The identification of putative prophages in the pangolin Pf genome was performed using the PHAST (PhAge Search Tool) with default thresholds (Zhou et al., 2011). PHAST uses a web server to perform a series of database comparisons and phage feature identification analyses, locating and annotating prophage sequences.

### Comparative virulence gene analysis

To identify the potential virulence factors, the RAST-predicted protein-coding genes in the genome of pangolin Pf were BLAST searched against the Virulence Factors Database (VFDB) (Chen et al., 2016; Chen et al., 2005) using the BLASTP of the BLAST software package (Altschul et al., 1997). In-house developed Perl scripts were used to screen out the genes that were identified as orthologous to virulence genes with at least 40% sequence identity and at least 40% sequence coverage in query and subject from the BLAST search. The filtered results were then further processed using in-house developed R scripts for clustering and constructing a graphical heat map with dendrograms, sorted according to similarities across the strains and genes.

**Table 1:** Genome statistics. The table shows the number of contigs, protein-coding genes, RNA, GC content, and virulence factor (VF) of pangolin Pf and members of the Paraburkholderiales and Burkholderiales.

**Strain name**	**Genome size**	**No. contigs**	**GC (%)**	**CDS**	**RNA**	**tRNAs**	**Subsystems**	**VF**
*P. fungorum* Pf	7.7	3	62.2	6,746	82	18	513	186
*P. fungorum* ATCC BAA-463	8.6	3	61.8	8,010	81	18	527	189
*P. phytofirmans* PsJN	8.1	2	62.3	7,562	81	19	517	192
*P. xenovorans* LB400	9.7	3	62.6	9,196	80	18	564	187
*P. phenoliruptrix* BR3459a	6.8	2	63.6	6,327	80	18	507	175
*P. phymatum* STM815	6.1	2	62.7	5,864	79	18	491	130
*B. mallei* ATCC 23344	5.8	2	68.5	5,738	67	18	501	282
*B. multivorans* ATCC 17616	6.8	3	66.8	6,562	80	18	524	172
*B. cepacia* GG4	6.4	2	66.7	6,119	66	18	529	181
*B. thailandensis* MSMB59	6.7	2	67.7	6,496	69	–	505	273
*B. pseudomallei* K96243	7.2	2	68.1	7,053	71	18	525	281
*B. oklahomensis* EO147	7.3	2	66.9	7,122	69	18	517	274
*B. cenocepacia* AU 1054	7.2	3	66.9	6,821	85	18	542	208
*B. pyrrocinia* DSM 10685	7.8	3	66.5	7,337	85	18	541	222
*B. vietnamiensis* LMG 10929	6.8	3	66.9	6,325	84	18	532	178
*B. ubonensis* MSMB22	7.1	3	67.3	6,531	89	18	529	201
*B. glumae* LMG 2196 ATCC 33617	6.4	2	68.5	5,832	81	18	493	233
*B. gladioli* ATCC 10248	8.5	2	67.9	7,511	81	18	521	199

## Results

### Genome summary

**Figure 1:**
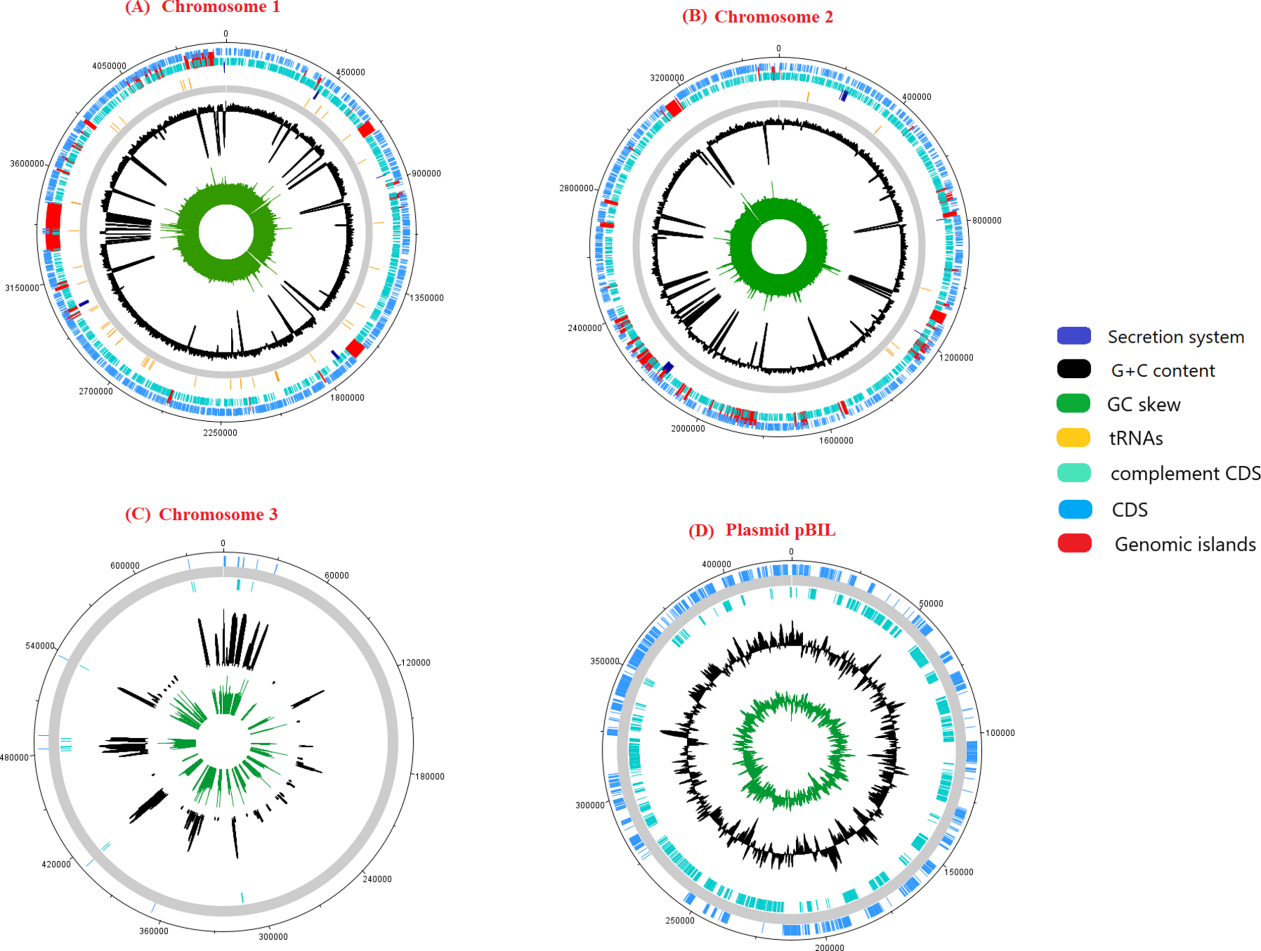
A schematic representation of the genome pangolin Pf. The ring represents chromosomes and plasmid of pangolin Pf, representatively (dark blue, secretion system; red, genomic island; yellow, tRNA; black, G+C content; green, GC skew). The outermost layer and second layer represent the forward and reverse coding region (CDS).

The genome sequence of pangolin Pf was assembled from the raw sequencing reads generated from the infected tissues (cerebrum and cerebellum) of a pregnant female pangolin and its infected fetal muscle (accession ID: SRR3949798, SRR3949787, SRR3949728, SRR3949765, and SRR11935558). The assembled genome of pangolin Pf has a genomic size of 7.7 Mbp (7,746,865 bp), covering 86% of the reference genome *P. fungorum* ATCC BAA 463 and with a GC content of 62.2%. The pangolin Pf genome had a total number of 222 contigs with an N50 metric of 69,666 bp (Table 1). The genome was plotted using a circular representation by including genomic features such as CDS, tRNA, GC content, and GIs (Fig. 1). When mapping the read sequences back to the assembled genome sequence, they covered 99% of the genome with an average read depth of 29X, suggesting the high quality of the assembled genome sequence and its suitability for downstream analyses.

### Confirmation of the taxonomic position of the pangolin Pf

**Figure 2:**
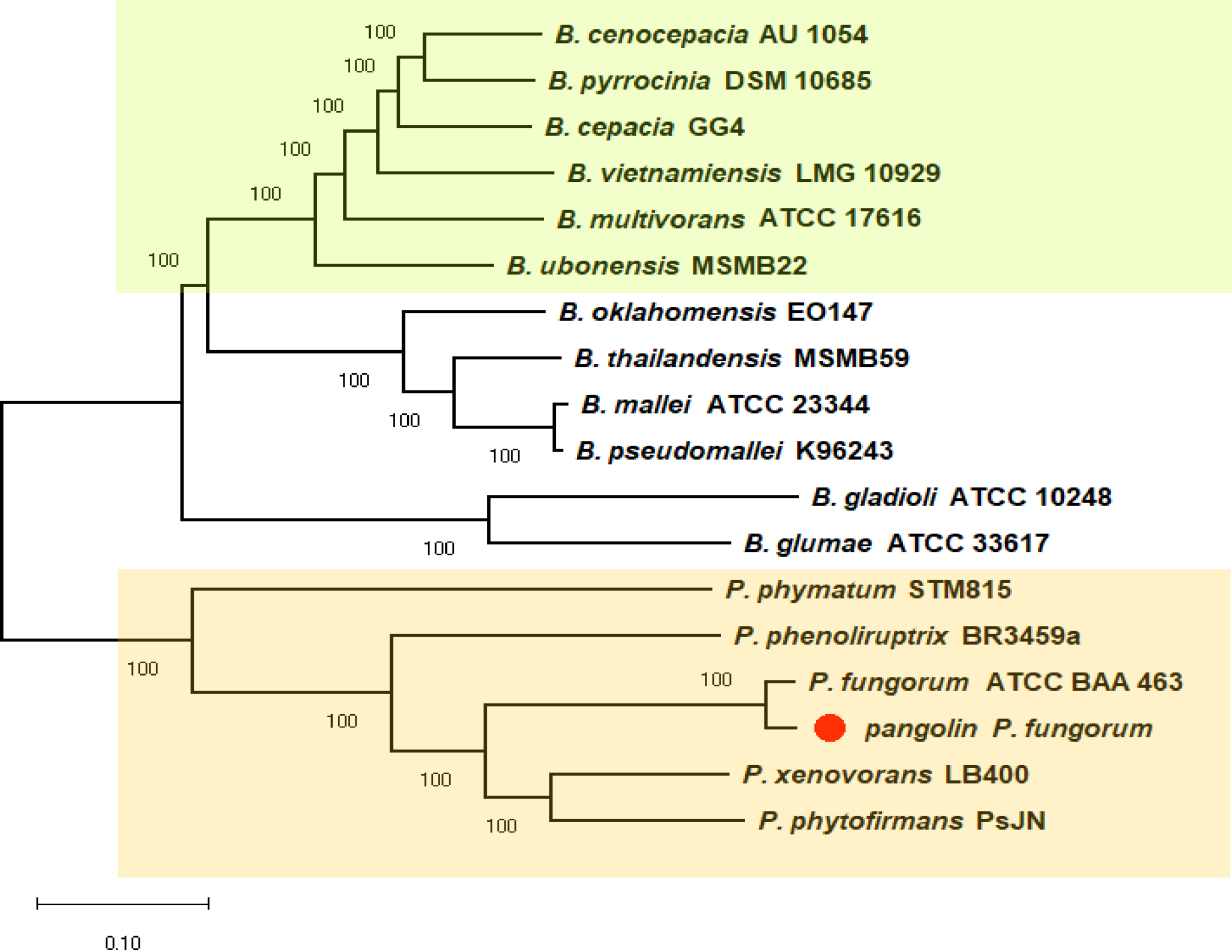
A core-genome SNP-based phylogenetic tree of pangolin Pf and closely related members of the Burkholderiales. The phylogenetic tree was generated using the Neighbour-joining algorithm method. Bootstrap numbers were generated in 1,000 replicates. The pangolin Pf genome sequence was aligned with the genome sequences of 17 other *Burkholderia* and *Paraburkholderia* species and the SNPs located in the core genome (conserved genomic regions among all species) were extracted for alignment and tree reconstruction. The green color part represents the Bcc group while the orange color part represents the Paraburkholderia group.

We previously discovered the identity of the pangolin Pf based on the preliminary analyses using two bacterial classification markers such as *rec* A and 16S rRNA genes (Fig. S1). The single marker gene approach may not be able to clearly differentiate all closely related members of Burkholderiales (Jin et al., 2020). Therefore, we used a more robust and reliable whole-genome method or the core-genome SNP approach to further confirm the identity of the pangolin Pf (Fig. 2). The core-genome SNP-based phylogenetic tree classified the pangolin Pf as *P. fungorum* species.

**Figure 3:**
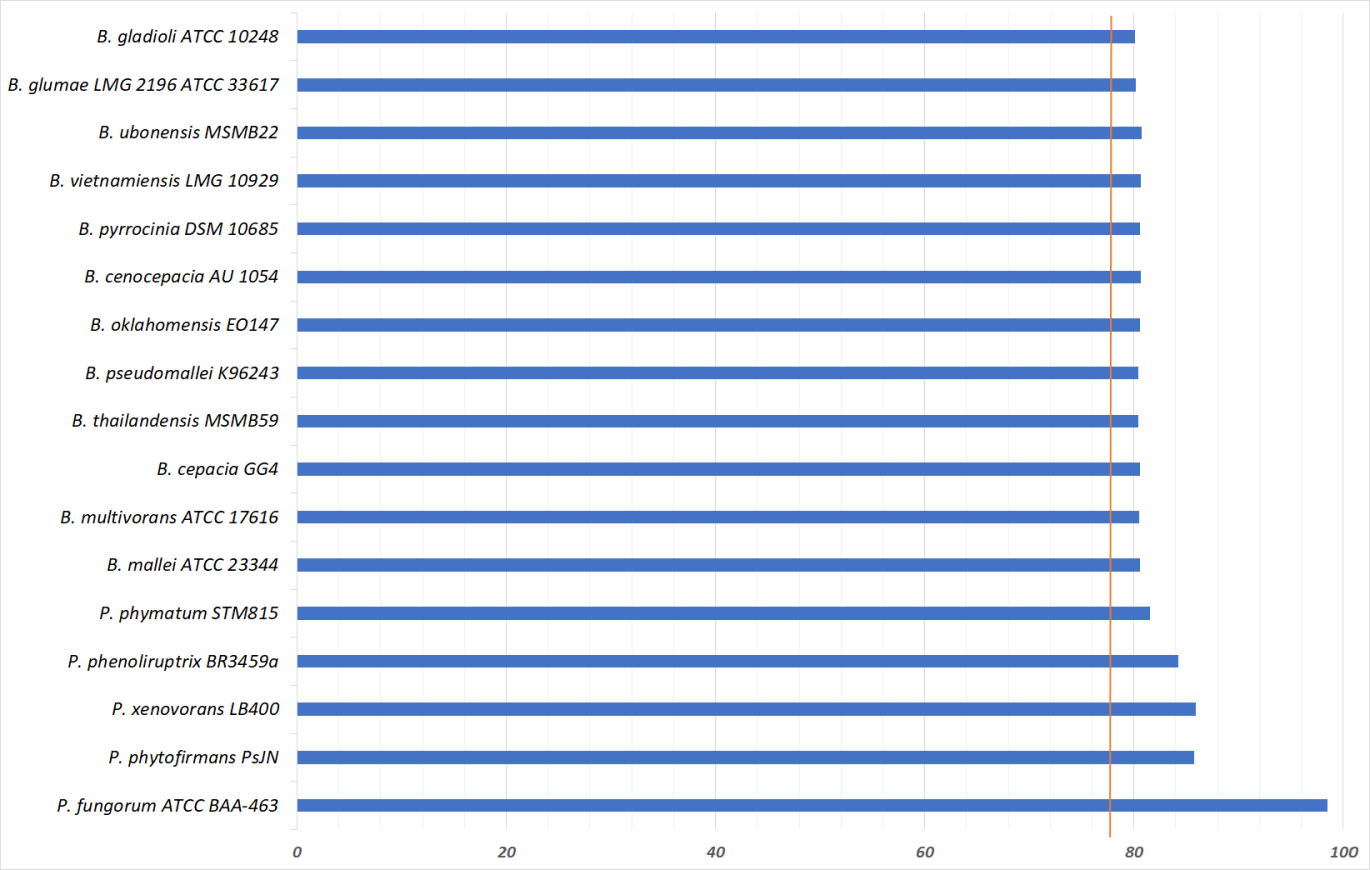
Average Nucleotide Identity statistics.

To further validate the taxonomic position of pangolin Pf, the average nucleotide identity (ANI) was calculated based on the method proposed by Goris and his colleagues to measure the relatedness between the members of the Burkholderiales and Paraburkholderiales (Goris et al., 2007). Using the nucleotide sequence of pangolin Pf as the reference genome, the ANI was calculated by performing a pairwise comparison between the reference genome with the genome of other members of Burkholderiales and Paraburkholderiales (Table 1). ANI results indicated that our pangolin Pf strain is closely related to *P. fungorum* ATCC BAA-463 with an ANI value of 98.49% (passing the threshold of 97% to define a species), whereas other species showed ANI values below the threshold (Fig. 3). Taken together, the results of our ANI and the core-genome SNP-based phylogenetic analyses are consistent and confirm that pangolin Pf is indeed *P. fungorum*.

### Functional analysis

**Figure 4:**
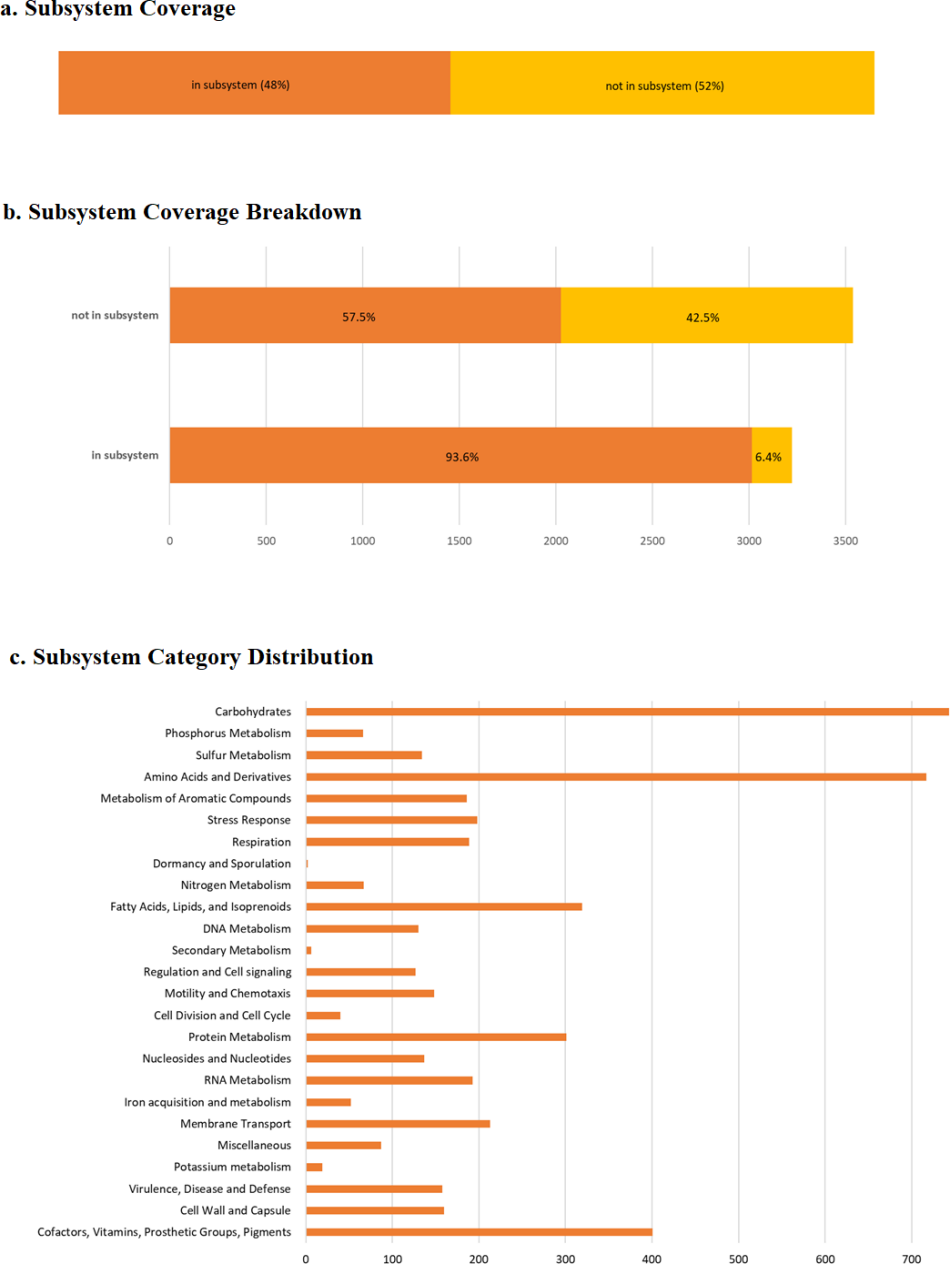
RAST annotation summary. (A) The subsystem coverage; (B) the subsystem coverage breakdown; (C) RAST annotation result showing the distribution of annotation across defined structural and functional subsystem roles in percentage. RAST uses a subsystem approach for annotation as assigning similar functional or structural roles into a group. For pangolin Pf, 48% of annotated genes belong to an identified functional role, or subsystem. The coverage breakdown shows the percentage of hypothetical and non-hypothetical annotations for genes assigned to subsystems and those for which a known functional role was unassigned.

RAST predicted 6,746 protein-coding genes and, 82 RNAs (Table S1). As anticipated, a large number of genes were enriched in basic functions such as carbohydrates (746 genes), amino acid and derivative metabolism (718 genes), cofactors, vitamins, prosthetic groups and pigments (401 genes), fatty acids, lipids and isoprenoids (318 genes), protein metabolism (300 genes), membrane transport (213 genes), and RNA metabolism (193 genes), which are important for bacterial survival (Fig. 4). We also observed 196 genes related to stress responses that may play an important role in bacterial adaptation to various environments (Table 2) (Koh & Roe, 1995; Koh & Roe, 1996; May et al., 1989).

Besides that, we identified 158 putative genes related to virulence, disease and defense including resistance to antibiotics and toxic compounds (132 genes), the host cell invasion and intracellular resistance (14 genes) and the ribosomally synthesized antibacterial peptides (12 genes), probably helping bacteria to inhibit the growth of similar or closely related bacterial strain (bacteriocins).

### Toxin-antitoxin (TA) systems

The genome of pangolin Pf has two putative toxin-antitoxin (TA) systems; *higAB* and *ygiUT*. The *ygiT* is the transcriptional repressor while *ygiU* has been predicted to be a cyanide hydratase induced upon biofilm formation, acting as a global regulator controlling biofilm formation by inducing motility (Shah et al., 2006). The TA gene has been suggested to play a role in mediating growth arrest during stress situations (Butt et al., 2013; Fivian-Hughes & Davis, 2010). Both *higAB* and *ygiUT* were located closely with each other, forming the toxin-antitoxin system.

**Table 2:** A list of RAST-predicted stress response genes in pangolin Pf. Stress response genes were predicted and categorized into heat shock, cold shock, detoxification, osmotic stress, oxidative stress, and periplasmic stress.

**Type of Stress Response**	**Predicted genes**
**Heat shock**	Chaperone protein DnaJ, DnaK, GroEL & GroES
	YggX a Probable Fe(2+)-trafficking protein
	Glutathione synthetase
	Heat shock protein GrpE, YegD, YciM precursor
	Hsp20 family protein
	Heat-inducible transcription repressor HrcA
	Nucleoside 5-triphosphatase RdgB, RpoH, tmRNA-binding protein SmpB, RpoE
	Translation elongation factor LepA
**Cold shock**	CspDG
**Detoxification**	DedA protein, Sulfate and thiosulfate import ATP-binding protein CysA (EC 3.6.3.25)
	Various polyols ABC transporter, ATP-binding component, periplasmic substrate-binding protein and permease component 1 & 2
**Osmotic stress**	Betaine aldehyde dehydrogenase (EC 1.2.1.8)
	Choline dehydrogenase (EC 1.1.99.1)
	Choline-sulfatase (EC 3.1.6.6)
	GbcA Glycine betaine demethylase subunit A
	High-affinity choline uptake protein BetT
	HTH-type transcriptional regulator BetI
	L-proline glycine betaine ABC transport system permease protein ProV (TC 3.A.1.12.1), ProW (TC 3.A.1.12.1) & transporter protein ProX (TC 3.A.1.12.1)
	Sarcosine oxidase alpha, beta, delta & gamma subunits (EC 1.5.3.1)
	Integral membrane protein YggT)
	Osmotically inducible proteins C & Y
	*proVWX* operon
**Oxidative stress**	Genes related to Glutathione Biosynthesis and gamma-glutamyl cycle
	Glutathione Non-redox reactions;
	Cluster containing Glutathione synthetase; Glutaredoxins
	Folate-dependent protein for Fe/S cluster synthesis/repair in oxidative stress;
	NTP pyrophosphohydrolases including oxidative damage repair enzymes
	Genes for Paraquat-inducible protein A & B
**Periplasmic stress**	HtrA protease/chaperone protein
	Outer membrane protein H precursor; sensor protease DegQ, serine protease
	Sigma factor RpoE negative regulatory protein RseA & RseB precursor
	Survival protein SurA precursor (Peptidyl-prolyl cis-trans isomerase SurA) (EC 5.2.1.8)
	*RpoE*, *RseA* and *RseB* of the *rpoE-rseABC* operon

### Urease

The pangolin Pf genome has several putative genes important for the urea decomposition and urease subunits such as Urea ABC transporter *urtBCDE*, Urease accessory proteins *ureDEFG*, Urease alpha, beta, and gamma subunits in the genome of pangolin Pf. The Urea ABC transporter *urtBCDE* involves in the uptake of urea and, in response to nitrogen limitation (Beckers et al., 2004). Urease, a nickel containing metalloenzyme, is a virulence factor enabling bacteria to survive by hydrolysing urea as the sole nitrogen source in nutrient limiting conditions to ammonia (Lin et al., 2012). For example, in other Gram-negative bacteria it has been shown that urease enables survival of the bacteria in strong acidic conditions of the stomach by neutralizing gastric acid with the released ammonia and thus playing a major role in the pathogenesis of gastroduodenal diseases (Cussac, Ferrero & Labigne, 1992).

### Stress response

There were 196 putative stress response genes identified in the pangolin Pf genome (Table 2). They were mostly related to oxidative stress, osmotic stress, detoxification, heat shock, cold shock, various polyols ABC transporter, ATP-binding component, periplasmic substrate-binding protein, and permease component. A large number of these stress response genes are associated with osmotic stress such as the ones related to Choline and Betaine Uptake and Betaine Biosynthesis. The *proVWX* operon has been reported to be remarkably stimulated in response to hyperosmotic stress encoding for a high affinity transport system for glycine-betaine (Kudva et al., 2016; Price et al., 2008). The glycine-betaine have a role as a cryoprotectant that help bacteria to stabilize their cell membranes and adapt to cold environments (Subramanian et al., 2011).

Several oxidative stress-related genes were also identified in the pangolin Pf genome. For instance, the presence of oxidative stress-related genes for Paraquat-inducible (Pqi) protein A and B (O’Grady & Sokol, 2011). These genes are the members of the *soxRS* regulon (Koh & Roe, 1995) and their expression can increase during the carbon or phosphate starvation in the bacterial stationary phase (Koh & Roe, 1996).

The pangolin Pf also had genes related to periplasmic stress such as *rpoE*, *rseA*, *rseB,* and *htrA* (Table 2). RpoE is an important transcription factor, which functions as effector molecules responding to extracytoplasmic stimuli and is essential for *Burkholderia* to cope with thermal stress (Vanaporn et al., 2008). Besides that, *htrA* is a virulence factor in *B. cenocepacia* important for the growth under the exposure to stress and survival *in vivo* (Flannagan et al., 2007).

### Membrane transport

The pangolin Pf genome has putative genes encoding ABC transporters related to membrane transport such as Phosphonate ABC transporter phosphate-binding periplasmic component; Oligopeptide ABC transporter, periplasmic oligopeptide-binding protein OppA, Oligopeptide transport system permease protein OppC & OppB, ATP-binding protein OppD & OppF; Branched-chain amino acid transport ATP-binding protein LivF & LivG, permease protein LivH & LivM); Dipeptide transport system permease protein DppB & DppC, ATP-binding protein DppD & DppF; Hopanoid-associated RND transporter, HpnN; Phosphonate ABC transporter permease protein phnE. The OppABC functions in the recycling of cell wall peptides (Goodell & Higgins, 1987). While the DppBCDF operon encodes an ABC transporter responsible for the utilization of di/tripeptides in *Pseudomonas aeruginosa* (Pletzer et al., 2014). The LivH and LivM permeases and the LivG and LivF ATPases are essential, to mediate the transport of these branched-chain amino acids into the cytoplasm (Adams et al., 1990). We also identified genes related to the resistance to antibiotics and toxic compounds such as the RND efflux system, the inner membrane transporter CmeB, outer membrane lipoprotein CmeC & NodT family; Cobalt/zinc/cadmium efflux RND transporter, membrane fusion protein, CzcB & CzcC family; Membrane fusion protein of RND family multidrug efflux pump, as well as the genes related to Ton and Tol transport systems in the pangolin Pf genome. The CmeABC gene is crucial to maintain a high-level resistance to fluoroquinolones and contributes significantly to the emergence of fluoroquinolone-resistant mutants (Yan et al., 2006). The RND multidrug efflux system is mainly responsible for the intrinsic multidrug resistance in Gram-negative bacteria (Guglierame et al., 2006).

### Secretion systems

In Gram-negative bacteria, the components of secretion systems are highly specialized macromolecule nanomachines secrete substrates across bacterial inner and outer membranes (Abby et al., 2016). The secreted substrates are responsible for a bacterium’s response to its environment and in physiological processes such as survival, adhesion, adaptation, and pathogenicity (Costa et al., 2015; Duangurai, Indrawattana & Pumirat, 2018). In the pangolin Pf genome, we identified several putative secretion systems which will be discussed below (Table 3).

**Table 3:** Secretion systems predicted in the RAST subsystem of the pangolin Pf genome.

**Type of secretion system**	**Predicted genes**
Type I Secretion System (T1SS)	Type I secretion outer membrane protein, TolC precursor
	Type I secretion system, outer membrane component LapE
Type II Secretion System (T2SS)	Secretion pathway protein CDEFGHIJKLMN
Type III Secretion System (T3SS)	Type III secretion bridge between inner and outermembrane lipoprotein (YscJ,HrcJ,EscJ, PscJ)
	YscU, SpaS, EscU, HrcU, SsaU, homologous to flagellar export components
	YscT, HrcT, SpaR, EscT, EpaR1, homologous to flagellar export components
	YscS, homologous to flagellar export components
	YscR, SpaR, HrcR, EscR, homologous to flagellar export components
	YscQ, homologous to flagellar export components
	Type III secretion cytoplasmic protein (YscL)
Type IV Secretion System (T4SS)	Type II/IV secretion system ATP hydrolase TadA/VirB11/CpaF, TadA subfamily
	Type II/IV secretion system ATPase TadZ/CpaE, associated with Flp pilus assembly
Type V Secretion System (T5SS)	Channel-forming transporter/cytolysins activator of TpsB family;
	ShlA/HecA/FhaA family
Type VI Secretion System (T6SS)	Proteins ImpA, ImpB, ImpC, ImpD;
	Protein of avirulence locus ImpE, ImpF, ImpG/VasA, ImpH/VasB, ImpI/VasC;
	Type VI secretion lipoprotein/VasD, ImpJ/VasE;
	Outer membrane protein ImpK/VasF, OmpA/MotB domain;
	Protein phosphatase ImpM;
	VgrG protein;
	ClpB protein;
	IcmF-related protein;
	Sigma-54 dependent transcriptional regulator/VasH

#### Type II secretion system (T2SS)

Many Gram-negative bacteria use this secretion system as the pathway to translocate proteins from the periplasm across the outer membrane. This system is normally used to secrete a variety of toxins and enzymes by pathogenic bacteria (Cianciotto & White, 2017). This system has been suggested to be the key, in making it possible for *Burkholderia Cepacia Complex* to function as opportunistic pathogens (Somvanshi et al., 2010). We identified several genes encoding the general secretion pathway proteins (general secretion pathway protein CDEFGHIJKLMN) in the pangolin Pf genome, increasing the possibility that the bacteria species could function as an opportunistic pathogen.

#### Type III secretion system (T3SS)

This secretion system plays an important role in the secretion of virulence factors by Gram-negative pathogens and the translocation of “effector” proteins into eukaryotic host cells (Saier, 2006). There are three classes in T3SSs in which class 1 and class 2 are predicted to mediate interactions with plants, however, class 3 (*bsa* locus) has been implicated in animal pathogenesis (Teh et al., 2014). Interestingly, we found the presence of BsaX in the pangolin Pf genome. The *bsa* (or *Burkholderia* Secretion Apparatus) found in *B. pseudomallei* plays an important role in helping the pathogen to survive and replicate in mammalian cells (Haraga et al., 2008). It also plays important roles in invasion, endosome escape and net intracellular replication in cultured cells and in virulence in murine and Syrian hamster models of melioidosis (Burtnick et al., 2008; Stevens et al., 2004; Stevens et al., 2002; Warawa & Woods, 2005).

#### Type V secretion system (T5SS)

This secretion system encompasses the auto-transporting and two partner systems (Henderson et al., 2004). In the pangolin Pf, a channel forming β-barrel transporter proteins belonging to the TpsB family (Jacob-Dubuisson, Locht & Antoine, 2001) was identified in a genomic island, suggesting that it might be acquired from other source. TpsB plays an important role as channel for the translocation of the exoproteins across the outer membrane and a specific receptor for TpsA signal secretion.

#### Type VI secretion system (T6SS)

It appears in a phage-tail-spike-like injectisome that has a potential to introduce effector proteins directly into the cytoplasm of host cells (Filloux, 2013). This secretion system has emerged as virulence factor that may take part in the pathogenic bacterial-host interactions or promote commensal or mutualistic relationships between bacteria and eukaryotes or to mediate cooperative or competitive interactions between bacteria (Jani & Cotter, 2010). For instance, in Gram-negative bacteria, it has been used for the purpose of interbacterial competition in Bcc member (Spiewak et al., 2019), and endure the innate immune response in host (Lennings, West & Schwarz, 2019). We also identified several regulatory genes that form the major requirement for a functional T6SS, such as structural proteins (VasH, VasK, VasF, VasA), effector protein (VgrG), and functional domains (chaperone ClpB, ImpA, ImpB, ImpC, ImpD, ImpF, ImpG, ImpH, ImpJ) in the pangolin Pf genome. The T6SS is comprised of the proteins encoded by *imp* locus and its counterparts which appear to have an important role in pathogen-symbiont host interactions (Records, 2011).

### Comparative virulence gene analysis

**Figure 5:**
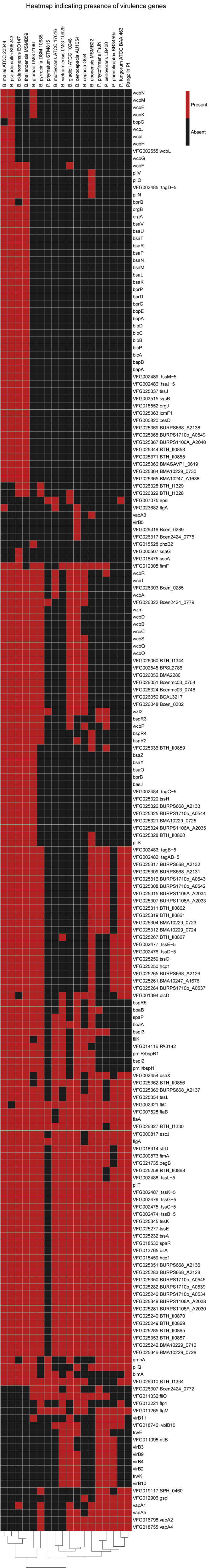
Comparative virulence gene analysis. Putative virulence genes across different members of the Burkholderiales and Paraburkholderiales strains as shown in graphical format - heatmap. The selected strains are shown as a horizontal line at the end of the map (*y*-ais) while the occurring virulence genes across the selected strains shown as a vertical line on the top of the map (*x*-axis). Red represents the presence of the virulence gene, whereas and black represents the absence of virulence gene.

To have a better understanding and more complete insights into the virulence of pangolin Pf, we performed a comparative virulence gene analysis of the genomes with known pathogenic *Burkholderia* sp. and *Paraburkholderia sp...* Our analysis showed that pangolin Pf shared some common virulence genes with these pathogenic *Burkholderia* strains such as *epsI, pilQ, bsaX*, *escS,* and *hrcS* (Fig. 5 and Table S2). Most of these predicted genes were associated with secretion systems especially Type III, and VI. The pangolin Pf also showed the presence of Type IV pilli (pilABCDERT) which are involved in adhesion (Piepenbrink & Sundberg, 2016). We found another set of common genes (eg. *bsaX, escS, and hrcS*) among the strains related to flagella that are involved in motility and invasion. Type III Secretion System is the main secretion system that mediates the secretion of effector molecules directly into host cells in *B. pseudomallei* and *B. mallei* (Beeckman & Vanrompay, 2010). Besides that, we found a T6SS cluster (TssA-TssM) in the pangolin Pf genome, probably playing an important role in bacterial competition (Spiewak et al., 2019).

### Genomic Island and prophage analysis

We wondered whether the pangolin Pf had acquired the horizontally transferred Genomic Islands (GIs) over the evolutionary time. GIs are clusters of genes that are inserted into a bacterial genome during a single horizontal gene transfer event, playing important roles in microbial evolution, virulence, drug resistance and/or adaptation to different environments (Zhang et al., 2014). To examine this, we predicted the GIs in the pangolin Pf genome using the IslandViewer4 (Bertelli et al., 2017). We identified putative GIs predicted in the pangolin Pf genome such as harboured genes related to Molybdenum cofactor biosynthesis (MoaD and MoaE), Molybdenum transport proteins (*modABCE*) and Molybdopterin biosynthesis protein *moeA* (Table S3). The molybdopterin biosynthesis pathway is important for the molybdopterin cofactor syntheses, which are required for a variety of molybdoenzymes such as nitrate reductase that which plays an important role in the denitrification process in bacteria under oxygen limiting conditions (Leimkühler & Iobbi-Nivol, 2016). The nitrate reductase and the Molybdopterin biosynthetic pathway have also been associated with bacterial virulence (Andreae, Titball & Butler, 2014; Filiatrault et al., 2013; Fritz et al., 2002). In addition, we identified T6SS genes in the GIs of pangolin Pf, suggesting that these genes might have originated from other sources.

There are three incomplete prophages predicted in the pangolin Pf genome, in region 1 with about 7.8 kb in length with 10 coding regions; region 2, 8.2 kb in length with 10 coding regions; and region 3 with 8.8 kb in length and with 6 coding regions (Fig. S2; Table S4), suggesting that pangolin Pf might have acquired these prophages a long time ago. No intact prophages or recently evolved complete prophages were detected in the genome. We cannot rule out the possibility that the secretion systems of pangolin Pf might provide a strong defense to the bacterium in preventing the integration of phages into its genome.

## Discussion

Here we present the genome sequence and comprehensive analysis of a *Paraburkholderia* species which was discovered from normally sterile tissue of a female pregnant Malayan pangolin. Our analyses showed that the pangolin Pf was supported by evidence from single-gene phylogenetic analyses, the reliable and robust core-genome SNP-based phylogenetic analysis and the ANI analysis. Genome analysis revealed genes involved in stress responses such as cold shock, detoxification, heat shock, and osmotic stress, and oxidative stress, and periplasmic stress. Since *P. fungorum* is considered an environmental bacterial species (Compant et al., 2008), these genes may play important roles to help the bacterial species to adapt and survive in a variety of environments.

Interestingly, the pangolin Pf genome also showed a number of genes related to virulence and defence mechanisms such as genes associated with maintenance of cell wall integrity, resistance to antibiotics and toxic compounds, invasion and intracellular resistance, which might make it more resistant to drugs. Moreover, we found that the genome of pangolin Pf consists of the secretion system proteins that may involve in pathogenicity. For instance, T3SSs are important for virulence in many Gram-negative pathogens (Coburn, Sekirov & Finlay, 2007). Furthermore, the T6SS has recently emerged as a critical virulence factor for the members of the Burkholderiales including the well-known pathogens *B. pseudomallei*, *B. mallei*, and *B. thailandensis* (Schwarz et al., 2014). We cannot rule out the possibility that the presence of the putative secretion systems in the pangolin Pf genome could render this environmental bacterium to a pathogen.

The *Paraburkholderia fungorum* that we studied here was found in an infected female pangolin, which is a placental mammal. Since we found this bacterium in the supposedly sterile tissues of pangolin, it is possible that the *P. fungorum* may have also the ability to colonize and infect humans. Moreover, we found that *P. fungorum* has a set of common virulence genes with the known pathogenic *Burkholderia* spp., raising the possibility that it may have the potential of being an opportunistic pathogen and cause human diseases especially this bacterium has also previously been isolated in the cerebrospinal fluid (Coenye et al., 2001), and the synovial tissue of humans (Loong et al., 2019). Our study raises caution about the use of *P. fungorum* for biocontrol and bioremediation in agriculture. However, more experimental studies are needed to further validate its pathogenic potential.

## Conclusion

In this study, we report the first comprehensive whole-genome analysis of pangolin Pf that was isolated from a Malayan pangolin. Here our study has provided new insights into the functions, secretion systems and virulence of *P. fungorum*. Besides that, the addition of the genome sequence provides a useful resource for future comparative analysis and functional work of *P. fungorum.*

##  Supplemental Information

10.7717/peerj.9733/supp-1 Supplemental Information 1 The phylogenetic tree constructed using a single marker gene The phylogenetic tree constructed for the pangolin Pf and closely related members of the Paraburkholderiales and Burkholderiales using single marker gene 16s rRNA and recA gene. The phylogenetic tree was generated using the Neighbour-joining algorithm method. Bootstrap numbers were generated in 1,000 replicates.

10.7717/peerj.9733/supp-2 Supplemental Information 2 Three incomplete prophage predicted These incomplete prophages consist of hypothetical protein, tail shaft, tail fiber, transposase, other phage like protein, and others.

10.7717/peerj.9733/supp-3 Supplemental Information 3 List of statistics of RAST information

10.7717/peerj.9733/supp-4 Supplemental Information 4 List of the protein percent identity and coverage of virulence gene for all genome

10.7717/peerj.9733/supp-5 Supplemental Information 5 Genomic Islands predicted by IslandViewer in the genome of pangolin Pf

10.7717/peerj.9733/supp-6 Supplemental Information 6 Three putative incomplete putative prophages details identified using PHAST

## Additional Information and Declarations

### Data Availability

The following information was supplied regarding data availability: Data is available at GenBank: CP028829–CP028832.
