# Supplementary figures and images for: Comprehensive genome analysis of a pangolin-associated Paraburkholderia fungorum provides new insights into its secretion systems and virulence
Source: PeerJ. 2020 Sep 3;8:e9733. doi: 10.7717/peerj.9733 (PMC7474880; doi:10.7717/peerj.9733)

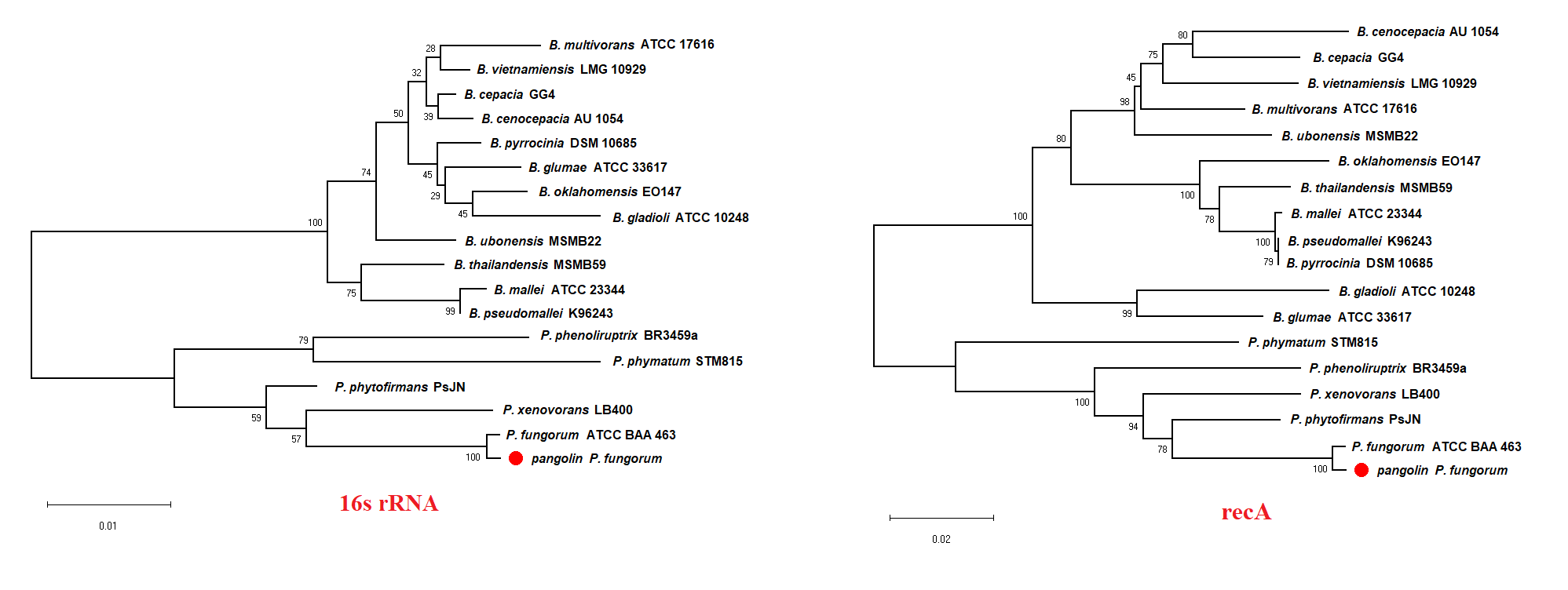

Supplement: Supplemental Information 1 — The phylogenetic tree constructed for the pangolin Pf and closely related members of the Paraburkholderiales and Burkholderiales using single marker gene 16s rRNA and recA gene. The phylogenetic tree was generated using the Neighbour-joining algorithm method. Bootstrap numbers were generated in 1,000 replicates. [file peerj-08-9733-s001.png]

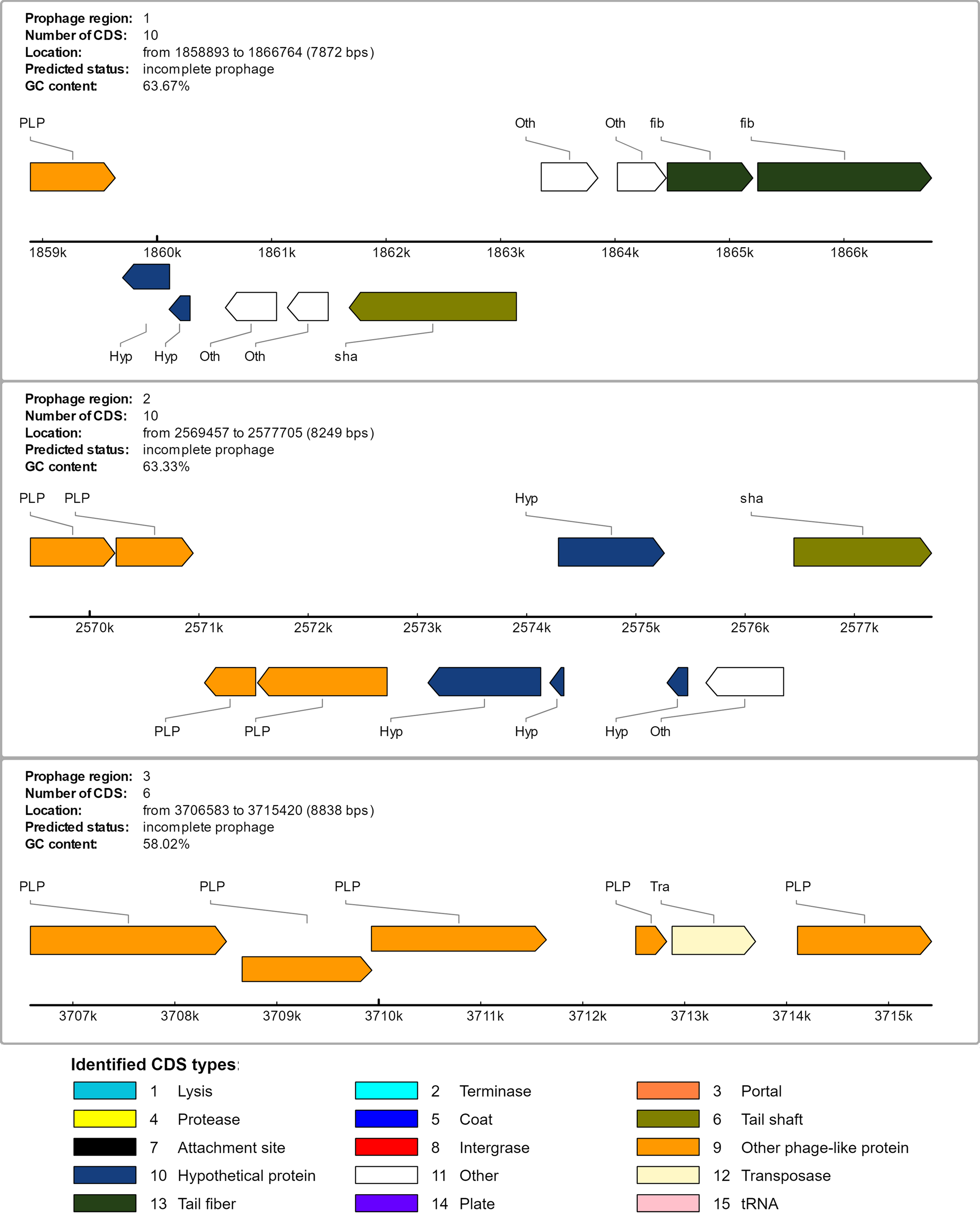

Supplement: Supplemental Information 2 — These incomplete prophages consist of hypothetical protein, tail shaft, tail fiber, transposase, other phage like protein, and others. [file peerj-08-9733-s002.png]
