# Supplementary material for: Comprehensive genome analysis of a pangolin-associated Paraburkholderia fungorum provides new insights into its secretion systems and virulence
Source: PeerJ. 2020 Sep 3;8:e9733. doi: 10.7717/peerj.9733 (PMC7474880; doi:10.7717/peerj.9733)
Supplement: Supplemental Information 3 [file peerj-08-9733-s003.docx]

| **Strain name** | **tRNAs** | **Subsystems** | **Secretion system** | **TA system** | **Urease** |
| --- | --- | --- | --- | --- | --- |
| *P. fungorum* Pf | 18 | 513 | II,V,VI | 4 | 7 |
| *P. fungorum* ATCC BAA-463 | 18 | 527 | II,V,VI,VIII | 4 | 7 |
| *P. phytofirmans* PsJN | 19 | 517 | II,V,VI,VII | 7 | 7 |
| *P. xenovorans* LB400 | 18 | 564 | II,III,V,VI,VIII | 4 | 6 |
| *P. phenoliruptrix* BR3459a | 18 | 507 | II,V,VI | 2 | 7 |
| *P. phymatum* STM815 | 18 | 491 | II,V | - | 7 |
| *B. mallei* ATCC 23344 | 18 | 501 | II,III,V,VI,VII | 7 | 7 |
| *B. multivorans* ATCC 17616 | 18 | 524 | I,II, VI,VII | 7 | 7 |
| *B. cepacia* GG4 | 18 | 529 | II,VI,VII | 10 | 7 |
| *B. thailandensis* MSMB59 | - | 505 | II,VI,VII | 5 | 7 |
| *B. pseudomallei* K96243 | 18 | 525 | II,III,V,VI,VII | 1 | 7 |
| *B. oklahomensis* EO147 | 18 | 517 | II,V,VI,VII | 4 | 7 |
| *B. cenocepacia* AU 1054 | 18 | 542 | II,VI,VII | 11 | 7 |
| *B. pyrrocinia* DSM 10685 | 18 | 541 | II,VI,VII | 17 | 7 |
| *B. vietnamiensis* LMG 10929 | 18 | 532 | II,V,VI,VII | 5 | 7 |
| *B. ubonensis* MSMB22 | 18 | 529 | II,V,VI,VII | 12 | 7 |
| *B. glumae* LMG 2196 ATCC 33617 | 18 | 493 | II,V,VI,VII | 2 | 7 |
| *B. gladioli* ATCC 10248 | 18 | 521 | II,V,VI,VII | 3 | 7 |

**(TA system : Toxin-antitoxin system)**

| **Strain name** | **Stress response** | | | | | |
| --- | --- | --- | --- | --- | --- | --- |
|  | **Heat shock** | **Cold shock** | **Detoxification** | **Osmotic stress** | **Oxidative stress** | **Periplasmic stress** |
| *P. fungorum* Pf | 16 | 6 | 22 | 38 | 90 | 7 |
| *P. fungorum* ATCC BAA-463 | 16 | 6 | 23 | 40 | 101 | 8 |
| *P. phytofirmans* PsJN | 16 | 6 | 32 | 46 | 99 | 7 |
| *P. xenovorans* LB400 | 16 | 7 | 23 | 51 | 105 | 8 |
| *P. phenoliruptrix* BR3459a | 17 | 6 | 21 | 47 | 69 | 7 |
| *P. phymatum* STM815 | 17 | 4 | 20 | 34 | 84 | 7 |
| *B. mallei* ATCC 23344 | 16 | 3 | 21 | 27 | 75 | 8 |
| *B. multivorans* ATCC 17616 | 17 | 4 | 24 | 37 | 89 | 8 |
| *B. cepacia* GG4 | 17 | 6 | 19 | 34 | 97 | 7 |
| *B. thailandensis* MSMB59 | 18 | 4 | 21 | 35 | 75 | 7 |
| *B. pseudomallei* K96243 | 16 | 4 | 23 | 30 | 86 | 8 |
| *B. oklahomensis* EO147 | 18 | 5 | 21 | 36 | 81 | 7 |
| *B. cenocepacia* AU 1054 | 16 | 6 | 27 | 36 | 103 | 7 |
| *B. pyrrocinia* DSM 10685 | 16 | 6 | 24 | 42 | 97 | 8 |
| *B. vietnamiensis* LMG 10929 | 17 | 5 | 23 | 33 | 92 | 10 |
| *B. ubonensis* MSMB22 | 17 | 5 | 22 | 30 | 92 | 7 |
| *B. glumae* LMG 2196 ATCC 33617 | 17 | 6 | 18 | 28 | 79 | 8 |
| *B. gladioli* ATCC 10248 | 16 | 5 | 19 | 52 | 110 | 8 |

| **Strain name** | **Virulence, Disease and Defense** | | |
| --- | --- | --- | --- |
|  | **Bacteriocins, ribosomally synthesized antibacterial peptides** | **Resistance to antibiotics and toxic compounds** | **Invasion and intracellular resistance** |
| *P. fungorum* Pf | 12 | 132 | 14 |
| *P. fungorum* ATCC BAA-463 | 13 | 189 | 14 |
| *P. phytofirmans* PsJN | 12 | 140 | 14 |
| *P. xenovorans* LB400 | 11 | 159 | 14 |
| *P. phenoliruptrix* BR3459a | 12 | 97 | 16 |
| *P. phymatum* STM815 | 11 | 109 | 14 |
| *B. mallei* ATCC 23344 | 10 | 94 | 14 |
| *B. multivorans* ATCC 17616 | 12 | 134 | 15 |
| *B. cepacia* GG4 | 11 | 135 | 13 |
| *B. thailandensis* MSMB59 | 12 | 109 | 14 |
| *B. pseudomallei* K96243 | 10 | 115 | 16 |
| *B. oklahomensis* EO147 | 11 | 119 | 14 |
| *B. cenocepacia* AU 1054 | 13 | 162 | 14 |
| *B. pyrrocinia* DSM 10685 | 15 | 145 | 14 |
| *B. vietnamiensis* LMG 10929 | 10 | 134 | 14 |
| *B. ubonensis* MSMB22 | 14 | 136 | 14 |
| *B. glumae* LMG 2196 ATCC 33617 | 10 | 105 | 14 |
| *B. gladioli* ATCC 10248 | 10 | 161 | 14 |
